# Circulating HMGB1 and RAGE as Clinical Biomarkers in Malignant and Autoimmune Diseases

**DOI:** 10.3390/diagnostics5020219

**Published:** 2015-06-16

**Authors:** Christin Pilzweger, Stefan Holdenrieder

**Affiliations:** 1 Klinikum Traunstein, Cuno-Niggl-Str. 3, Traunstein 83278, Germany; E-Mail: christinpilzweger@gmail.com; 2 Institute of Clinical Chemistry and Clinical Pharmacology, University Hospital Bonn, Sigmund-Freud Str. 15, Bonn 53127, Germany

**Keywords:** HMBG1, RAGE, circulation, biomarkers, prognosis, therapy response, tumor, autoimmune disease, DAMP

## Abstract

High molecular group box 1 (HMGB1) is a highly conserved member of the HMG-box-family; abundantly expressed in almost all human cells and released in apoptosis; necrosis or by activated immune cells. Once in the extracellular space, HMGB1 can act as a danger associated molecular pattern (DAMP), thus stimulating or inhibiting certain functions of the immune system; depending on the “combinatorial cocktail” of the surrounding milieu. HMGB1 exerts its various functions through binding to a multitude of membrane-bound receptors such as TLR-2; -4 and -9; IL-1 and RAGE (receptor for advanced glycation end products); partly complex-bound with intracellular fragments like nucleosomes. Soluble RAGE in the extracellular space, however, acts as a decoy receptor by binding to HMGB1 and inhibiting its effects. This review aims to outline today’s knowledge of structure, intra- and extracellular functions including mechanisms of release and finally the clinical relevance of HMGB1 and RAGE as clinical biomarkers in therapy monitoring, prediction and prognosis of malignant and autoimmune disease.

## 1. Introduction

Circulating biomarkers are of growing importance in the difficult fields of prognosis, prediction and therapy monitoring in cancer disease [1,2]. Even though many biomarkers have been proven to have immunomodulating abilities [3,4], their use in autoimmune diseases has become a subject of interest, too [5,6].

In malignant disease, an approach to individualized therapy is highly desirable and ever more therapy options increase the need for risk stratification for and therapy monitoring during cytotoxic therapy. To date, clinical criteria are the most important indicators used for clinical decision making, along with standardized evaluation criteria based on medical imaging, *i.e*., RECIST-criteria [7]. However, these criteria only apply for solid tumors and all imaging techniques have their flaws, as e.g., sonography is strongly dependent on experience of the investigator. Further, alterations of the surrounding tissue like inflammation and irradiation after-effects can impede exact measurements even in computed tomography (CT) or magnetic resonance tomography (MRT) [8,9]. The most reliable diagnostic measure so far was histological examination, however sample collection is invasive, not appropriate for serial testing and with a relatively high chance of missing some parts of a heterogeneous tumor [10]. This applies also to the new molecular tests in tumor tissue that are used to stratify patients to specific targeted therapies, such as EGFR mutations as indicators for gefitinib and erlotinib therapies in lung cancer or KRAS as exclusion criterion for cetuximab therapy in colorectal cancer [11,12].

This “diagnostic gap” of missing reliability in data for clinical decision making could be filled by circulating biomarkers. They are easily obtained by venipuncture, cost-effective and allow an evaluation of ongoing changes in disease activity or therapy response by serial testing [2,13]. In many tumor entities, oncological biomarkers are used in everyday clinical diagnostics, e.g., PSA in prostate cancer or CEA in colorectal cancer [14,15,16]. Recently, a multitude of promising molecules has come up by research in molecular structures, genomics and metabolic pathways, but only few of them have been proven to be of clinical use and were integrated in clinical diagnostics such as human epididymis protein 4 (HE4) and progastrin related peptide [17,18].

However, even if a potential marker on its own fails to show statistically relevant improvement of current diagnostics, it may well be of value as part of diagnostic panels [19].

## 2. Structure

### 2.1. HMGB1

HMGB1 is a member of the HMG-box family, abundantly expressed and present in virtually all human cells. Initially known as a nuclear structure protein with DNA binding and bending functions, its role as a chaperon in cellular replication, transcription, recombination and DNA-repair was revealed during the last years. However, not only its nuclear or intracellular functions have become subject of interest—the latest findings display its multiple extracellular interactions with effects on the immune system, autoimmune disease, inflammation and tissue homeostasis. The correlation of extracellular HMGB1 levels and immune activity respective disease activity could make HMGB1 a useful tool for diagnosis, disease monitoring and evaluation of therapy response.

HMGB1 consists of two DNA-binding subunits, termed A-box and B-box, and a negatively charged acidic tail, necessary for special intra- and intermolecular interactions with e.g., histones or nucleosomes [20,21,22,23]. The two boxes of HMGB1 bind non-sequence dependent, but with special affinity to distorted, bent or kinked DNA and can induce structure modulation as bending or uncoiling [22,24]. While the A-box plays a major role in recognition of DNA-damage, the B-box is suspected to bear the proinflammatory sequence, as only the B-box was shown to form an immune-activating complex with CpG-A-oligodeoxynucleotides (ODNs) [25,26].

The genetic sequence of HMGB1 is located on chromosome 13q12 and consists of approx. 10,000 base pairs, encoding for 214–216 amino acids, resulting in a molecular weight of about 25 kD [27,28]. Although the tertiary structure of the two boxes is well known, the complete structure of HMGB1 is difficult to define as the instability needed for its many interactions leads to a multitude of possible conformations [29]. Expression is regulated by a multitude of different expression factors, such as p53 and c-myc, known for their important role in cell death and tumor growth [30,31]. Posttranslational acetylation, phosphorylation or methylation lead to nuclear activation and distribution to various cell compartments [32,33]. Because of the generally observed extremely high intracellular HMGB1 levels—about one million copies in most cells [34]—it was initially considered to be a housekeeping gene, but expression varied enormously within and between cell types reviewed in [35]. The intracellular concentration of HMGB1 differs widely in different tissues with expression rates varying up to 100-fold between high- and low-expressing tissues [36]. Interestingly, expression negatively correlates with differentiation, supporting the idea of HMGB1 as an excellent biomarker for dedifferentiated malignant tissues [35,37].

### 2.2. RAGE

RAGE (receptor for advanced glycation end products) is a type I transmembrane protein and member of the immunoglobulin superfamily. RAGE is highly expressed in alveolar type I and II cells in the lungs [38], but is also present in most other tissues including heart, kidney, liver, brain, endothelium of blood vessels as well as smooth and skeletal muscle [39,40,41,42]. It is increasingly expressed when potential ligands such as HMGB1 or inflammatory mediators are abundantly expressed, e.g., in cardiovascular disease, diabetes or cancer [40,43,44,45]. The genetic sequence encoding RAGE is found on chromosome 6 near MHC class II and the genes encoding TNF-α [46]. The cell surface 45–50 kDa protein consists of 404 amino-acids [47], forming three extracellular domains (one variable “V”, and two constant “C1 and C2”), a single transmembrane domain and a cytoplasmic, hydrophobic tail [48]. As a pattern recognition receptor, membrane-bound RAGE is able to interact with a multitude of different ligands. Apart from HGMB1 and S100, the most well-known binding partners, it binds to extracellular DNA, promoting its uptake into endosomes and facilitating its immune recognition via TLR-9. DNA-RAGE interaction is achieved in a sequence-independent manner via the sugar-phospate backbones and leads to conformational change in RAGE, including formation of higher-order receptor complexes [49].

**Figure 1:**
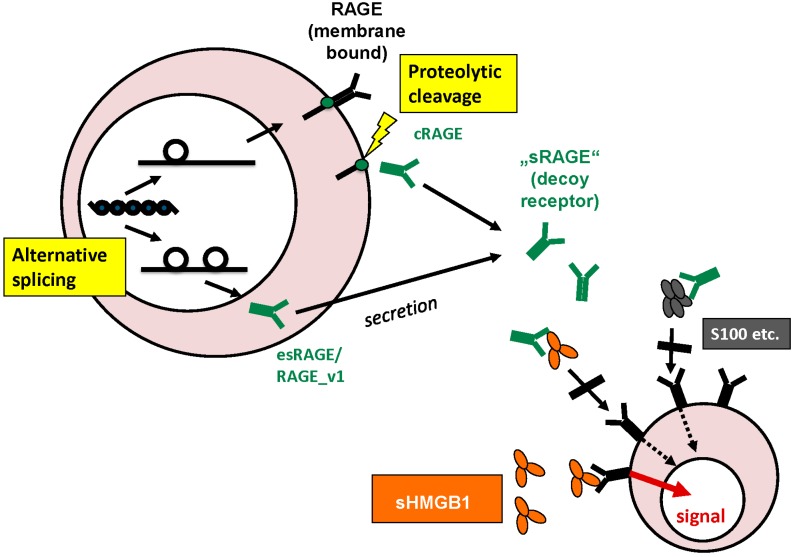
Origin and functions of sRAGE: sRAGE can derive from alternative splicing or proteolytic cleavage of the outer membrane domain of the full-length transmembrane receptor. In the extracellular space, sRAGE can act as decoy receptor for HMGB1 and other ligands.

In addition to the membrane-bound form of full-length RAGE, the extracellular domain on its own as soluble or sRAGE was detected in the extracellular space and blood circulation. This extracellular domain of RAGE only consists of an *N*-terminal signal peptide and three immunoglobulin domains, one V-type and two C-type domains. Ligands binding to RAGE were first shown to happen at the V-type domain [50], the latest results, however, report some ligands binding to the C-type domains [51]. sRAGE can derive from proteolytic cleavage of the extracellular part of the transmembrane receptor, called cleaved or cRAGE [52] or rather from alternative splicing of RAGE. Matrix metalloproteases were shown to be involved in ectodomain shedding of sRAGE [53]. Alternative splicing can lead to altered extracellular domain variants, mostly around the V-domain important for ligand binding, or either to soluble forms of which RAGE_v1 (RAGE splice variant 1) formerly called esRAGE (endogenous secretory RAGE), or simply sRAGE, was shown to be the only secreted form. Alterations in the reading frame during alternative splicing with introduction of 16 new amino acids allow for specific antibody-based measurement of RAGE_v1/esRAGE. Thus RAGE_v1 was found to be the second most prevalent isoform after full-length RAGE. sRAGE has become object of scientific interest as it can act as a decoy receptor for HMGB1 and is easily obtained as a serum biomarker by venipuncture [54,55,56] (Figure 1).

## 3. Physiological Functions and Cellular Release

The functions of HMGB1 and RAGE can each be divided into (intra-) cellular and extracellular functions. HMGB1 exerts many of its functions by activation of membrane-bound RAGE. To avoid repetitions, the emphasis of this section is put on HMGB1.

### 3.1. Intracellular Functions of HMGB1

Intracellular functions of HMGB1 were originally revealed within the nuclear compartment as a DNA-binding and -bending chaperon. There, HMGB1 mediates essential nuclear processes, e.g., transcription, recombination, replication and repair. By its intrinsic function of chromatin bending, HMGB1 can approximate distant regulatory sequences and improve the binding of transcription factors to certain gene promoters [57]. Characteristic amino acid sequences at the C-terminal tail of HMGB1 seem to have a major role in stimulation of transcription [23], however the tail may have regulatory functions in general, as Stros *et al*. showed lower activation of topoisomerase IIα gene promoter by native HMGB1 in comparison to modified HMGB1 without its tail [58]. HMGB1 enhances the binding affinity of many sequence-specific proteins to DNA usually by direct binding reviewed in [57] but also activates transcription by direct binding to nucleosomes [59] or interaction with TATA-box complex proteins [60]. Among the selection of transcription factors influenced by HMGB1 are also tumor suppressor factors p53 [61] or p73 [62] and NF-κB [63,64]. *In vitro*, HMGB1 was shown to influence the sequence specific binding of HOX-proteins in a dose dependent manner by protein-protein interaction [65]. Binding of HMGB1 to retinoblastoma protein results in cell growth suppression [66].

In recombination, HMGB1 promotes DNA-bending for bridging spacers of different length in V(D)J-recombination and stimulates cleavage and RAG protein binding to spacer signals [67]. Nucleosome sliding is improved by chromatin remodeling of distorted DNA, leading to enhanced binding affinities of ACF, a nucleosome remodeling complex [59].

For replication of parvoviruses, HMGB1 is an important cofactor for a strongly bent replication origin called “rolling-hairpin” [68]. However, *in vitro* HMGB1 has inhibiting effects on replication, unless the native protein is modified by acetylation, phosphorylation or elimination of the C-terminal tail [69].

With its strong affinity for bent and distorted DNA, HMGB1 is strongly qualified for detecting and remodeling damaged chromatin structure, like double strand breaks, and is directly involved in histone deacetylation [70,71]. The improvement of nucleosome sliding is also important in DNA repair, as it provides access to damaged DNA sections for chromatin remodeling factors and repair proteins. Furthermore, HMGB1 can facilitate recognition of DNA damage by certain repair proteins which bind with a higher affinity to hooked and distorted DNA [72,73]. By complex-binding to repair proteins, HMGB1 was shown to accelerate nucleotide excision repair (NER) by coordination or induction of NER proteins [73,74,75]. Through modification of base excision repair (BER) by protein interaction with correlating enzymes, HMGB1 plays an important role in maintenance or loss of genomic stability. Stimulation of preferred long-patch BER leads to genomic maintenance while stabilization of intermediate DNA-structures or CAG repeats can lead to development of tumor cells or neurodegenerative processes [73] In a pancreas-specific HMGB1-deficient mouse model, intracellular HMGB1 limited nuclear damage and nucleosome release, leading also to milder clinical symptoms in acute pancreatitis [76]. HMGB1 further demonstrated to be vital in sustaining nuclear homeostasis and inducing stress responses like autophagy in a study on HMGB1 global knockout mice [77].

One mechanism of regulation of apoptosis *vs.* autophagy is the protection of autophagy proteins becil1 and ATG5from calpain-mediated cleavage by cytosolic HMGB1, inhibiting the formation of proapoptotic fragments [78]. HMGB1’s translocation from the nucleus to the cytosol can be induced by a variety of signals such as activated poly(ADP)-ribose polymerase (PARP-1) after alkylating DNA damage [79], in human dendritic cells after infection with dengue fever [80] or in alveolar macrophages by FIP200, an autophagy initiating protein, after infection with pseudomonas aeruginosa [81]. In activated monocytes, cytosolic HMGB1 is acetylated and phosphorylated, inhibiting its resumption into the nucleus and thus leading to cytoplasmic accumulation [82,83].

### 3.2. Cellular Release of HMGB1

**Figure 2:**
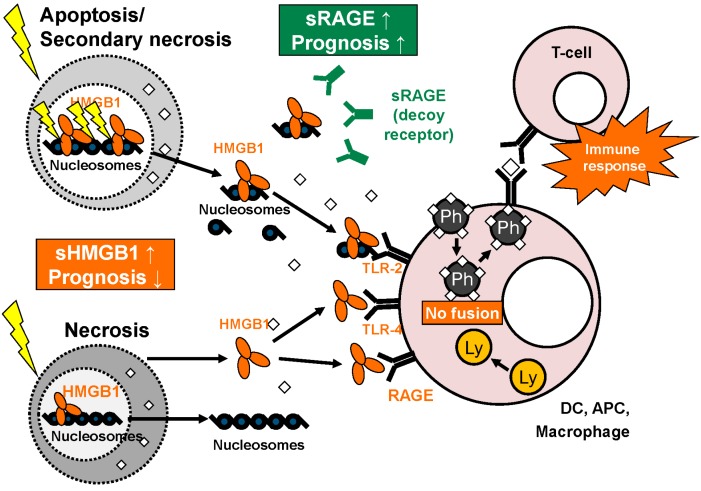
Overview: functions of HMGB1 and sRAGE. HMGB1 is released as single molecule during necrosis while it is set free as complex bound to nucleosomes in apoptosis/secondary necrosis. In the extracellular space, HMGB1 binds to various receptors like TLR-2, TLR-4 and RAGE on the surface of macrophages, dendritic cells (DC) and other antigen presenting cells (APC), leading to inhibited fusion of phagosomes and lysosomes within these cells. This, in turn, promotes presentation of phagocyted antigens with effective specific T-cell response. sRAGE derived from alternative splicing or proteolytic cleavage can act as a decoy receptor for HMGB1. As circulating biomarkers, high levels of HMGB1 and low levels of sRAGE correlate with poor prognosis (adapted from [103] with permission from De Gruyter).

HMGB1 is passively released from necrotic or damaged cells or actively secreted by cells of the immune system or tissue cells under hypoxic conditions reviewed in [84] (Figure 2). While passive HMGB1 release out of necrotic or damaged cells was known as “immunogenic” with subsequent activation of the immune system, apoptosis was suggested to be “immunological silent” as degradation happened in a physiological and regulated way and no significant HMGB1 release was detected [85]. However, in various cell types, measurable HMGB1 release was reported from apoptotic cells without signs of necrosis [86,87]. The blocking of autophagy in dying cells leads to intracellular retention of HMGB1 [88]. The redox state of extracellular HMGB1 seems to be an important factor, as the reduced form induces autophagy, while oxidized HMGB1 promotes apoptosis. These mechanisms play an important role in drug resistance and response to chemotherapy in malignant disease [89]. Regulation of the redox state is achieved by intracellular caspase activation and release of oxygen radicals [90]. During apoptosis HMGB1 stays closely linked to nuclear DNA and thus is released in complex with nucleosomes. This complex has immunogenic functions when binding to and activating the TLR-2 receptor [86,91]. Macrophages and dendritic cells actively release HMGB1 after stimulation by apoptotic cells [92], endotoxins, TNF or interleukins [93]. In dendritic cells and some other immune regulatory cells, HMGB1 is translocated into vesicles before release in a non-classical secretion pathway [94]. In hypoxic hepatocytes, active HMGB1 secretion was shown to be regulated by a TLR4- and ROS-dependent pathway involving inhibition of calcium/calmodulin-dependent kinases [95].

### 3.3. Extracellular Functions of HMGB1

**Figure 3:**
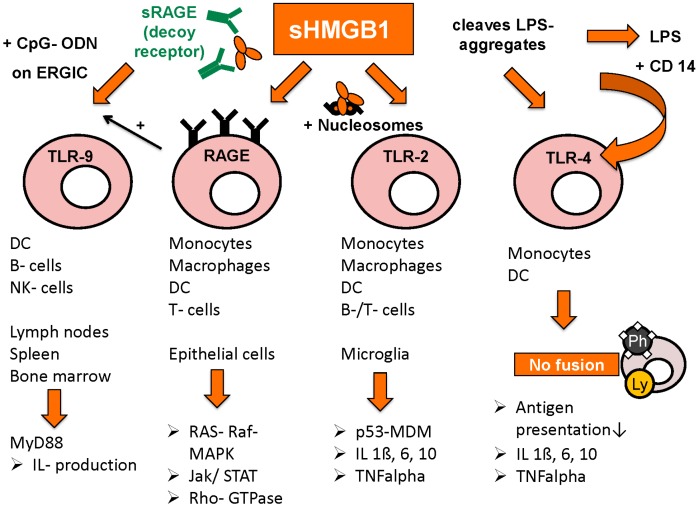
Receptors for HMGB1 and following pathways (adapted from [103] with permission from De Gruyter).

Once in the extracellular space, HMGB1 can bind to a multitude of receptors and exert its function as a danger associated molecular pattern (DAMP). By binding to its first identified and favourite receptor RAGE, HMGB1 can activate many different intracellular signal pathways such as RAS-RAF-MAP or Rb2F, important factors in regulation of transcription or cell cycle progression [96]. There are also reports on induction of Rho GTPases [97] and the Jak/STAT pathway [98]. The HMGB1-RAGE synergy is also important for the homing of immature dendritic cells to lymph nodes, where the development of TH1 type cells is especially promoted [99]. Complexes of nucleosomes and DAMPs like HMGB1 were shown to limit macrophage survival by RAGE-dependent activation of TNF-alpha release [100]. In the interaction of TLR-9 with single stranded oligonucleotides (CpG-ODNs) RAGE forms a complex with CpG-ODNs and HMGB1, leading to improved presentation of CpG-ODNs to TLR-9 and thus to accelerated cytokine production in a MyD88-dependent way [26,101]. Furthermore RAGE interacts in the binding of HMGB1 to TLR-2 or TLR-4 leading to activation of the p53-MDM-pathway, and there is manifold interaction between the three different receptor-dependent ways of activation [96]. RAGE may thus be a kind of “adapter” rather than a simple receptor [102] (Figure 3).

Toll-like receptors TLR-2, TLR-4 and TLR-9 are also important binding partners for HMGB1’s immunogenic functions, as well as interleukin receptor IL-1 and CD24.

Binding to TLR-2 is activated by HMGB1 in complex with nucleosomes, leading to increased release of proinflammatory cytokines IL-1beta, IL-6, Il-10, as well as augmented expression of MHC class II, TNF-alpha, costimulatory CD 86 and DC maturation marker CD 83. Release of HMGB1/nucleosomes complexes occurs most frequently in apoptosis or secondary necrosis, when HMGB1 is still bound tightly to chromatin. By contrast, HMGB1 derived from necrotic cells is rapidly detached from chromatin and rather acts solely as a danger associated molecular pattern (DAMP) [91].

TLR-4 activation is achieved by two different mechanisms: either by HMGB1 alone, as is the case in DCs where HMGB1-TLR-4 interaction was shown to promote antigen presentation by inhibition of antigen-digestion through fusion of phagosomes and lysosomes [104]. On the other hand, HMGB1 was shown to bind to and disintegrate aggregates of lipopolysaccharides (LPS), leading to complex-binding of lipopolysaccharides and CD14, which in turn leads to higher measurable amounts of TNF-alpha compared to stimulation by HMGB1 or lipopolysaccharides alone [105]. The intracellular mechanism however seems to be the same, LPS/CD14-complex also results in inhibited fusion of phagosomes and lysosomes in macrophages [106].

The promotion of TLR-9/CpG-ODN induced cytokine production via HMGB1/RAGE/CpG-ODN complexes was already mentioned above [26,101]. Moreover, HMGB1 increases IL-1 receptor activation by binding to IL-1beta [107]. In hepatocellular cancer, HMGB1 binds to mitochondrial DNA released from hypoxic cells and promote tumor cell growth by TLR-9 activation [108]. In general, HMGB1 seems to promote immune stimulation when complex-bound to proinflammatory co-molecules, whereas on its own, HMGB1 rather reduces immune response and enhances tissue repair processes [109]. Such host-protecting functions can be seen in improved discrimination between DAMPs and pathogen associated molecular patterns (PAMPs) via HMGB1-dependent activation of CD24-Siglec G pathway [110]. A certain micro-RNA, miR-34c, is differentially expressed in peripheral blood mononuclear cells depending on the presence of HMGB1 in the administered fluids. Further findings suggest that miR-34c leads to inflammasome activation in response to DAMPs, whereas other micro-RNAs were rather specifically expressed after administration of PAMPs [111]. In lupus nephritis, only extracellular but not intracellular HMGB1 was shown to induce pathologic immune response (macrophage activation) against self-DNA by promoting its accumulation in endosomes and TLR-9 activation [112].

### 3.4. Intracellular and Extracellular Functions of RAGE

The intracellular functions of RAGE are rather to be called membrane-bound functions, as RAGE is a transmembrane receptor with only a small intracellular part. Membrane-bound RAGE is found on the surface of various epithelial cells and cells of the immune system. Besides being the favourite receptor of extracellular HMGB1 and other advanced glycation end products (AGEs), it binds to and interacts with proteins of the s100-family, immunoglobulin light chains or amyloid, as well as chromatin structures DNA and RNA. Thus RAGE as a pattern recognition receptor (PRR) is part of signalling pathways in inflammation, autoimmunity disease or malignant processes, as shall be explicated in the following chapter.

Extracellular soluble RAGE, derived from alternative splicing or proteolytic cleavage, is able to bind to HMGB1 and impede its extracellular functions, *i.e*., act as a decoy receptor for HMGB1 [56]. Consistently, in a population of 626 healthy volunteers, HMGB1 and sRAGE correlated inversely in blood circulation [113]. sRAGE also acts as a decoy receptor for S100B, another ligand of its membrane-bound form [55]; however, its full range of extracellular functions is still a subject of investigation.

## 4. Pathophysiological Functions

### 4.1. Role of HMGB1 and RAGE in Malignant Disease

**Figure 4:**
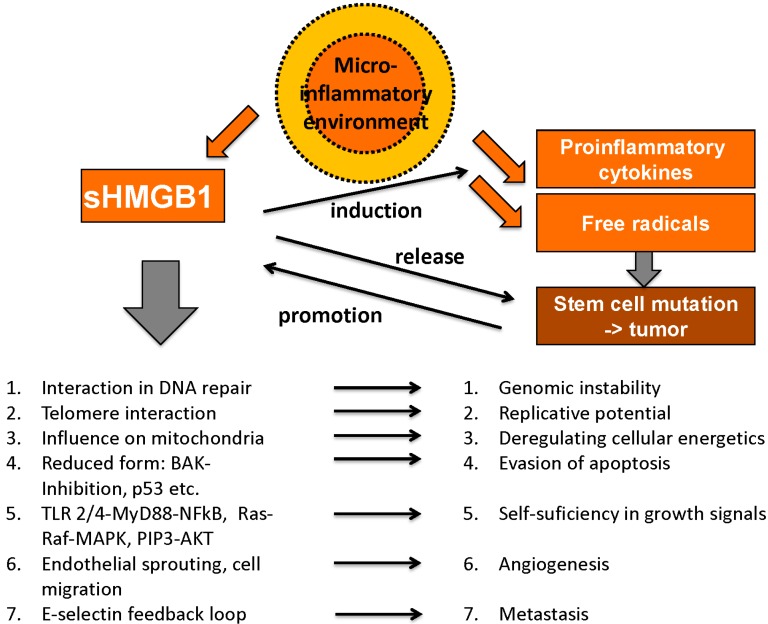
Tumor-promoting abilities of HMGB1 (adapted from [103] with permission from De Gruyter).

HMGB1 plays an important role in many processes related to tumor development and cancer growth; however, it exerts contrary functions that can be divided into pro- and anticancerogenic facets. Hanahan and Weinberg claimed six “hallmarks of cancer” as there are unlimited replicative potential, evasion of programmed cell death (apoptosis), self-sufficiency in growth signals, insensitivity to inhibitors of growth, ability to develop blood vessels (angiogenesis) and tissue invasion and metastasis [114,115]. Meanwhile, these have been expanded to ten hallmarks of cancer by addition of deregulating cellular energetics, avoiding immune destruction, genome instability and mutation and tumor-promoting inflammation [116]. Interestingly, HMGB1 is involved in each of these essential pro-tumorigenic processes (Figure 4).

#### 4.1.1. Unlimited Replicative Potential

Telomere length is the limiting factor for replicative potential in eukaryotic cells as telomeres are shortened each time during mitosis (due to the specific mode of operation of DNA polymerases) and the maximum number of mitoses usually restricts the lifetime of a cell. In the development of malignant tissue this control process is overcome by mechanism of telomere elongation [117]. As described above HMGB1 has a high affinity for bent and kinked DNA and was indeed shown to interact with a hairpin structure on a telomere of the parvovirus “minute virus of mice” [68].

#### 4.1.2. Genome Instability and Mutation

Being a DNA-modulating chaperone, HMGB1 can influence genome instability and mutation, another hallmark of cancer. HMGB1’s physiological functions include repair mechanisms to maintain genomic stability [73] and a loss of HMGB1 was shown to cause genome instability [118]. Furthermore, HMGB1 promotes expression of TopoisomeraseIIα and promotes its activity in DNA transcription with Rb having a regulatory impact on this stimulation [58]. However, other studies reported HMGB1 to inhibit nucleotid excision repair under certain circumstances [119] and to induce single-strand breaks and induction of CAG repeats, leading to genomic instability [73]. HMGB1’s role again seems to be two-faced and needs to be further examined.

#### 4.1.3. Evasion of Apoptosis

As damaged or mutated cells are subject to degradation by apoptosis, evasion of apoptosis is another essential step for tumor development. Even later abrogation of apoptotic mechanisms including contact inhibition is necessary for tumor growth. HMGB1 was shown to inhibit BAK-specific induction of apoptosis in yeast cells and prevent mammalian cells from cell death induced by ultraviolet radiation and cell death mediators CD95, TRAIL, Casp-8, and Bax [120]. The redox state of HMGB1 plays a pivotal role, as the only the reduced form was shown to prevent apoptosis in malignant tissue, whereas oxidized HMGB1 rather promotes apoptosis and can improve therapy response on systemic chemotherapy [89]. The reduced form of HMGB1 can again be divided into all-thiol-HMGB1 with only chemokine activity and disulfide-HMGB1 inducing cytokine activity. While disulfide-HMGB1 activates the nuclear factor NF-κB pathway and promotes production of proinflammatory cytokines like TNF-alpha and IL-6 in fibroblasts and macrophages, all-thiol-HMGB1 showed chemoattractant activity in fibroblasts [121,122]. HMGB1 was also called a “redox sensor” as its redox state defines its translocation, release and activity, thus reflecting the complex interaction between multiple pathways involved in stress response [123].

#### 4.1.4. Self-Sufficiency in Growth Signals

Self-sufficiency in growth signals as a characteristic feature of malignant tissue is achieved by mutations in the signal transductions chain, most of them mimicking external growth factor receptor activation [114]. HMGB1 induces MAP-kinases, which are important in signal chains regulating cell growth and survival, via RAGE activation and NF-κB, part of the same signal chains, via RAGE, TLR-2 or TLR-4 [115,124]. Furthermore, a HMGB1-dependent TLR-4/MyD88 pathway induces MAPK and Akt signaling chains, which end in inflammatory response and are also part of growth signaling in malignant cells, e.g., the phophatidylinositol 3 (PIP3)/Akt pathway is activated by HMGB1 in colon cancer cells [125,126].

#### 4.1.5. Insensitivity to Inhibitors of Growth

However, internal growth signals can be overruled by external inhibitors of growth; thus, insensibility towards these signals is another survival advantage for tumor tissue. Tumor cells can continuously enter G2-, synthesis (S-) and mitotic phases of the cell cycle, resulting in accelerated growth while normal cells enter the non-proliferative G0 phase when tissue homeostasis or a certain level of specialization is reached. *In vitro*, HMGB1 overexpression in MCF-7 cells leads to accelerated cell cycle progression when cells were stimulated by estrogen [127]. By contrast, interaction of HMGB1 with the antiproliferative mediator retinoblastoma protein (Rb) via an LXCXE motif-dependent mechanism was shown to promote cell cycle arrest and apoptosis [66]. Probably, the redox-state and/or the binding partners are again important decision points on the crossroad of diverse functions.

#### 4.1.6. Deregulating Cellular Energetics

Fast growing tumors need extensive supply of oxygen and nutrients. The change from combined oxidative and glycolytic metabolism to a preference of anaerobic lactate production for ATP-generation is widely known as the “Warburg effect”. Lately, Yang *et al*., showed PKM2 to regulate this effect and simultaneously promote HMGB1 release [128]. Moreover, HMGB1/RAGE interaction plays an important role in mitochondrial function in pancreatic tumor cells, including ATP production. There is also a positive feedback loop with extracellular HMGB1 promoting RAGE mitochondrial expression, which in turn leads to augmented mitochondrial complex I activity [129]. Transketolase-like 1 (TKTL1), an enzyme similar to transketolase was discovered in aggressive tumors, leading to rapid fermentation of glucose into lactate and thus inhibiting the formation of advanced glycation endproducts (AGEs), RAGE’s preferred ligands [130].

#### 4.1.7. Angiogenesis

However, despite all the intratumor metabolic changes, every tumor reaches a point, when the surrounding vascularization is not sufficient anymore for supply of oxygen and nutrients. Necrosis of central tumor parts occurs where diffusion cannot sustain cell metabolism and these hypoxic areas release angiogenetic growth factors like vascular endothelial growth factor (VEGF). Activation of macrophages by VEGF in turn leads to further release of angiogenetic cytokines like HMGB1, which was shown to promote endothelial sprouting *in vitro* [131]. Via TLR-4-activation, HMGB1 can induce corneal neovascularization [132]. There may be even a positive feedback mechanism of HMGB1 release and HMGB1-induced activation of several proinflammatory signal chains, ending in chronic inflammation and augmented angiogenesis [133]. Not only angiogenesis is promoted by HMGB1, the protein was also shown to dose-dependently induce sprouting of lymphatic vessels *in vitro*, possibly relevant for lymphatic metastasis [134].

#### 4.1.8. Metastasis and Tissue Invasion

HMGB1 and RAGE are further strongly involved in metastasis and tissue invasion. RAGE is preferentially expressed in poorly differentiated tumor cells of gastric cancer tissue and RAGE immunoreactivity also correlated with depth of invasion and lymph node metastasis. RAGE-positive cancer cells were mainly situated at sites of active invasion and in all lymphatic metastases [135]. Moreover, the HMGB1/RAGE interaction seems to involve the expression of p44/p42, p38 and SAP/JNK MAP kinases along with matrix metalloproteases (MMP), which are closely connected to kinase cascades [136]. TIAM1 (T lymphoma invasion and metastasis 1), a guanine nucleotide exchange factor activating RAC and other invasion-promoting molecules like Trophinin, induced increased HMGB1 expression as well [137,138]. An example for effective positive feedback loops including HMGB1, the metastasis-promoting factor E-selectin upregulates HMGB1 release to the extracellular milieu, which in turn leads to augmented expression of E-selectin [139]. Another way of supporting metastasis is reduction of immune defense. Indeed, HMGB1 release lead to decreased numbers of macrophages in lymph nodes and liver-specific antigen presenting Kupffer-cells [140,141]. By contrast, HMGB1/RAGE interaction was reported to diminish tumor cell migration *in vitro* and lead to decreased metastasis in a lung cancer model [45]. This may either be explained by different binding sites to RAGE (in this case via COOH-terminal motif), or perhaps by the lack of a very specific, modulating tumor environment in the human body.

#### 4.1.9. Microinflammatory Environment

Concerning the individual tumor environment, local inflammatory reactions in the so called “microinflammatory environment”, which is one of the new inflammatory hallmarks of cancer, play a major role [115,116]. With its manifold functions in tumor development as well as in immune response, HMGB1 represents the two-faced role of the immune system in general: while an inflammatory microenvironment promotes tumor growth, effective immune recognition results in the effective elimination of malignant cells [102].

Chronic inflammation is essential for tumor development, a thesis that is backed by various observations: tumors often arise from sites of chronic inflammation, immune cells can be found in almost all types of cancer, experimental blocking of inflammatory mediators (e.g., HMGB1) inhibits cancer development and administration of anti-inflammatory drugs (AID) was even reported to reduce the risk for certain tumor entities reviewed in [142]. HMGB1 exerts multiple proinflammatory functions in this tumor microenvironment, including activation and induction of proinflammatory cytokines (via NF-κB) [143], the interaction with cytokines like IL-1β [107] and the promotion of angiogenesis [132] and metastasis [135]. Once the release of free radicals and proinflammatory cytokines reaches a certain level, the inflammatory environment is able to sustain itself in positive feedback loops. Long-term presence of free radicals and mutagenic factors induces cell degradation and dedifferentiation and can thus initiate malignant processes. Tumor growth is then promoted by inflammatory mediators until the tumor tissue itself sustains inflammation by cytokine release and again various feedback loops [142]. Chronic lymphocytic leukemia (CLL) is a malignant disease strongly dependent on a promoting microenvironment including nurse-like cells (NLC). Extracellular HMGB1 releaed from CLL cells was shown to promote differentiation of NLCs in a time- and concentration-dependant manner via RAGE and TLR-9 [144].

##### Avoiding Immune Destruction

Whereas chronic low-grade local activation of immune system sustains and promotes tumor growth, an effective recognition of degraded cells with adequate immune response leads to tumor elimination. The key to the two different ways of response may be indicated by HMGB1: while the molecule is detected constantly on malignant tissues in low concentrations, massive release is reported after application of systemic chemotherapy. These high concentrations of HMGB1 over a relatively short period of time lead to effective activation of the acquired immune system, namely increased recruitment of dendritic cells (DC) that degrade engulfed apoptotic tumor cells, resulting in antigen presentation and specific T cell responses [102,145]. Augmented release of HMGB1 by tumor cells on cytotoxic treatment with Ad-TK (+ganciclovir) was even detected to be part of activating effective immune response against glioblastoma cells—a noteworthy fact, as usually the blood-brain barrier prevents immune response [146]. However, HMGB1 again plays a dual role as it was also shown to induce apoptosis in macrophage-derived dendritic cells, weakening the body’s anti-cancer immune response [147]. Furthermore, HMGB1 release from tumor cells can suppress naturally acquired CD8 T-cell antitumor response by promoting regulatory T-cells to produce immunosuppressive IL-10 [148].

All in all, HMGB1 was clearly shown to interact in manifold and sometimes diverse ways in tumor development. The different pro- and anti-tumor functions seem to be initiated and controlled by a certain “combinatorial cocktail” of substances present in the immediate surroundings of the tumor cell in certain amounts and probably released in a defined spatiotemporal sequence. This is a plausible reasoning as mediators of cell death and immune activation like ATP or HMGB1 are constantly and ubiqituously present in the human body, yet signal chains are only activated when necessary [149]. There is still a lot of research needed in this field to understand the complex interactions that also may intervene in therapeutical intentions.

While HMGB1 exerts many functions in promotion of tumor growth and was shown to be upregulated in tumor tissue early [150], RAGEv1 was reported to be downregulated in lung, prostate and brain tumors when compared to healthy control. Acting as a decoy receptor for RAGE ligand S100B, RAGEv1 impaired RAGE/ligand induced tumor formation, cell invasion, and angiogenesis *in vitro*. This is achieved by blocking of MAPK, p38 and JNK signaling. Tumor cells overexpressing RAGEv1 were altered in expression of gene classes important for the already mentioned hallmarks of cancer such as cell cycle progression, apoptosis, tissue invasion and metastasis. Mass of tumors with RAGEv1 expression was significantly decreased compared to wild-type tumors *in vitro* and in animal models [55].

### 4.2. Role of HMGB1 and RAGE in Autoimmune Disease and Non-Malignant Diseases

Through its immune modulating properties, HMGB1 plays an important role in autoimmune diseases like rheumatoid arthritis (RA) and systemic lupus erythematosus (SLE), as well as in acute inflammatory events like sepsis and trauma. The redox state of HMGB1 is important for intracellular decision on apoptosis or autophagy [89], however HMGB1 interferes also in other ways in induction of autophagy, e.g., by inducing transcription of HSPβ1, a regulator of actin cytoskeleton dynamics [151] or binding to beclin-1, leading to initiation and sustain of autophagy reviewed in [84,152]. Furthermore, it prevents beclin-1 from caspase-mediated degradation, resulting in no proapoptotic fragments and in induction of autophagy instead of apoptosis [78]. RAGE in turn is known as HMGB1’s favourite receptor in exerting its immune modulating functions and was also shown to facilitate DNA uptake into endosomes and presentation to TLR-9 for effective immune response resp. autoimmune response [49].

#### 4.2.1. Systemic Lupus Erythematosus

This might also be important in systemic lupus erythematosus (SLE), which is characterized by presence of characteristic auto-antibodies against double-stranded DNA (dsDNA) and histones. Degradation of cells physiologically leads to continuous release of small amounts of chromatin fragments like nucleosomes without any specific immune response, so in SLE, a lack of immune tolerance is assumed. Binding of HMGB1/nucleosome complexes to TLR-2 activates DCs and other immune cells, leading to production of specific anti-dsDNA and anti-histone antibodies, whereas nucleosomes on their own could not initiate a specific antibody response [91]. HMGB1 serum levels in SLE patients were increased, especially in those with renal involvement, and correlated positively with disease activity measured by SLEDAI scores and proteinuria, as well as with levels of anti-HMGB1 antibodies. The presence of HMGB1-specific antibodies suggests a pathogenetic role of HMGB1/anti-HMGB1 immune complexes in SLE [153]. Membrane-bound RAGE interacts in the pathogenesis of SLE vasculitis through HMGB1/RAGE mediated proinflammatory response to immune complexes in human endothelial cells, while sRAGE was again shown to attenuate the proinflammatory effects of immune complexes [154].

#### 4.2.2. Rheumatoid Arthritis

In rheumatoid arthritis (RA), a positive proinflammatory feedback loop was found, where HMGB1 induces release of TNF-alpha from activated macrophages, which in turn induces translocation to the cytosol in macrophages [155]. Lately, HMGB1 was shown to promote angiogenesis in RA by enhanced expression of vascular endothelial growth factor (VEGF) and HIF-1α activation [156]. Via binding to a RANKL-responsive sequence (receptor activator for NF-κB ligand), HMGB1 promotes osteoclastogenesis and thus an additional destructive effect on joint tissue in RA [157,158]. In a mouse model, intrathecal application of HMGB1 induced long-lasting hypersensitivity and activation of spinal microglia and astrocytes via TLR-4 [159].

By contrast, sRAGE levels correlated negatively with joint damage and vascular disease in patients suffering from rheumatoid arthritis, probably by acting as decoy receptor for proinflammatory RAGE ligands like S100 protein family members [160] Indeed, *in vitro* application of sRAGE to human-salivary gland cell lines before addition of proinflammatory S100A4 led to down-regulation of RAGE expression and inhibition of NF-κB activation [161].

#### 4.2.3. Acute Inflammation

In acute inflammatory events like SIRS or sepsis, HMGB1 plays an important role in the course of systemic immune response. *In vitro* and in a mouse model, administration of endotoxin led to release of HMGB1 from macrophages with measurably augmented extracellular levels. The same effect occurred after addition of TNF or IL-1 *in vitro* [93]. Endotoxin-mediated release of HMGB1 *in vitro* and in a mouse model seems to be dependent on C5l2, a receptor for complement factor C5a [162] and secretion from activated macrophages may involve inflammasome assembly and caspase-1 activation [163]. RAGE and TLR-9 probably play a major role during innate immune response as absence or blocking of these two receptors was protective for septic shock [164]. In SIRS, high plasma levels of HMGB1 were also shown to correlate with poor prognosis in a dog model [165]. Genetic polymorphisms of HMGB1 were mirrored in clinical parameters and prognosis also in humans with SIRS [166]. The ways of signal transduction of immune activation seem to be identical in sepsis and SIRS, involving complex interaction of the DAMP HMGB1 with surface receptors, leading to long-term maintenance of immune response [166,167]. In studies on trauma, HMGB1 levels were elevated in all kinds of trauma such as severe trauma [168,169], burn trauma [170] or iatrogenic trauma [171]; however, these alteration can derive from trauma-induced cell death (without any participation of the immune system) as well [172]. Various anti-HMGB1 treatments have been tested in inflammatory diseases including HMGB1 neutralizing antibodies, ethyl pyruvate, cholinergic agonists like nicotine, antagonists of endogenous HMGB1 and thrombomodulin with promising results [34].

In a mouse model with artificially induced sepsis by “cecal ligation and puncture” (CLP) procedure application of sRAGE-Fc fusion protein significantly reduced pulmonary inflammation score and mortality. Even the expression of proinflammatory cytokines was reduced in the treated group compared to untreated controls with measurable differences in IL-6 levels [173].

## 5. Methods for Detection of HMGB1 and sRAGE

HMGB1 can be detected by sandwich-ELISA (enzyme-linked immunosorbent assay), Western Blot or EMSA (electromobility shift assay).

Detection by ELISA is easy to perform, cost-effective and the favourite method for HMGB1 quantification in cell cultures; however, HMGB1 levels in serum and plasma were lower when compared to Western Blot. This may be due to masking factors like autoantibodies, lipopolysaccharides or IL1-beta, inhibiting HMGB1 recognition by sandwich-ELISA [174]. Pretreatment with perchloric acid leads to separation of these factors from HMGB1 and to higher measured amounts of HMGB1 [175]. However, it is unclear if the total amount of circulating HMGB1 is superior in clinical relevance, as masked HMGB1 may no longer exert its extracellular functions.

In Western Blot detection, the pretreatment of serum and plasma samples with SDS generally leads to solution of masking protein complexes, resulting in reliable total amounts of HMGB1. Background proteins are made visible as well and can be seen as indicators of test specificity [174,175,176]. In clinical routines, however, Western Blot is not as easily performed, takes more time and is more expensive than “common” ELISA.

The electromobility shift assay (EMSA) makes use of special binding capacities of HMGB1 to radioactively marked DNA sequences. The marked HMGB1/DNA complexes are then analyzed in electrophoresis, also leading to minimized background proteins. Work expense is similar to Western Blot, but preparation and provision of radioactive markers complicates detection in daily routines [176].

For the detection of sRAGE in serum and plasma samples, there are ELISA [177], Western Blot and EMSA [178] tests available. Advantages and disadvantages are similar to previously explained HMGB1 detection tests. However, as there are different isoforms, there are two different ELISA systems: sRAGE-detecting ELISAs assessing the total amount of free soluble RAGE (esRAGE and cRAGE) and a specific esRAGE ELISA with specific antibodies against its unique C-terminus sequence. For cleaved RAGE alone, there is no specific test available so far. Some studies have measured sRAGE levels with both systems and correlated the results [179,180], yet comparison between the two independent systems is still difficult.

## 6. Clinical Relevance of Circulating HMGB1 and sRAGE

### 6.1. Clinical Relevance of Circulating HMGB1 and sRAGE in Autoimmune Disease and Non-Malignant Disease

In SLE, HMGB1/nucleosome complexes derived from apoptotic cells can evoke characteristical anti-dsDNA and anti-histone immune globulin responses against self-DNA [91]. Ma *et al*., reported plasma levels of HMGB1 to significantly differ in active disease compared to healthy control. Furthermore, quantitative variations could be seen with highest HMGB1 levels in active disease [181]. Particularly in lupus nephritis, HMGB1 levels were shown to be significantly elevated, correlating also with clinical features like proteinuria and SLEDAI scores [153]. In a study on 116 children suffering either from SLE or juvenile idiopathic arthritis (JIA), all patients showed significantly higher HMGB1 serum levels and significantly lower sRAGE serum levels when compared to 27 healthy controls. High levels of HMGB1 also correlated with hepatosplenomegaly or serositis in systemic onset JIA. Another study on sRAGE on 105 SLE patients revealed significantly lower plasma levels of sRAGE in patients than in healthy controls [182].

In a rat model with artificially induced arthritis, HMGB1 was detected in all nuclear, cytoplasmic and extracellular compartments while it was restrained in the nucleus in healthy controls. Matching results were reported from patients suffering from rheumatoid arthritis (RA): measurable amounts of extracellular HMGB1 were detected in synovial fluid of 14 out of 15 patients [183]. Another study on rat and mouse models proved HMGB1 antibodies to reduce histologic severity of RA, correlating with the mean arthritis score [184]. Goldstein *et al*., found HMGB1 serum levels to be significantly elevated in RA patients compared to healthy controls, displaying also a correlation with the disease activity score DAS-28 [185].

Serum sRAGE levels in 138 patients suffering from rheumatoid arthritis were negatively associated with serum levels of C-reactive protein (CRP) and history of vasculitis. By determination of serum levels of S100 protein members, sRAGE was further shown to have opposite effects on S100 proteins backing its function as decoy receptor for RAGE ligands [160]. Another study on sRAGE levels in early rheumatoid arthritis including 94 patients under anti-inflammatory therapy reported sRAGE levels to be negatively correlated with arterial stiffness assessed by aortic augmentation index (Alx). sRAGE levels were significantly increased after one year of therapy, and in multivariate analysis, changes in sRAGE levels were independent predictors connected with a change in Alx [186]. In juvenile idiopathic arthritis, sRAGE levels were lower in patients’ sera compared to healthy controls, too. They were negatively correlated with erythrocyte sedimentation rate and swollen joint count. In stable disease, also sRAGE levels remained stable [187].

HMGB1 levels were also significantly elevated in antineutrophilic cytoplasmatic antibody (ANCA)-associated vasculitis with higher levels in active than in inactive disease or in remission phase, thus reflecting active inflammation. There was no difference between the subgroups of Wegener granulomatosis, microscopic polyangiitis and Churg-Strauss syndrome [188]. Furthermore, in 42 patients suffering from Behçet’s disease, HMGB1 serum levels were also significantly elevated compared to healthy controls, however, they did not correlate with disease activity [189].

In 50 children with acute Kawasaki disease, sRAGE levels were decreased in patients with high disease activity and in non-responders after intravenous immunoglobulin treatment, being inversely correlated to S100A12 [190]. These findings were lately backed by Qi *et al.*, who found sRAGE levels to be significantly lower in the acute stage of Kawasaki disease and increased in afebrile and subacute stages, again being negatively correlated with S100A12 and resistin [191].

Not only in autoimmune disease but also in acute inflammatory conditions like “Systemic Inflammatory Response Syndrome” (SIRS), HMGB1 levels are elevated, e.g., in patients after major gastrointestinal surgery of alimentary tract carcinoma, where serum peak levels also correlated with duration of SIRS and pulmonary dysfunction [192]. Cohen *et al*., reported elevated HMGB1 plasma levels as early as 30 min after severe trauma, with HMGB1 levels correlating with severity of injury, systemic inflammatory response, posttraumatic coagulation abnormalities and complement activation [168]. Lately, Wang *et al*. confirmed elevated HMGB1 levels in patients with blunt chest trauma with higher levels in patients, with multiple organ dysfunction syndrome and sepsis being also associated with poor outcome [169]. After burn trauma, HMGB1 plasma levels correlated with burned body surface and were shown to be generally elevated early after trauma, with significantly higher levels in non-survivors [170]. In all types of trauma, HMGB1 levels in circulation may only reflect cellular damage, but mirror active inflammation as well as HMGB1 exerts multiple proinflammatory functions [172].

sRAGE plasma levels were elevated within 30 min after severe trauma in humans, too, correlating with severity of injury, posttraumatic coagulopathies and endothelial cell activation. A significant relationship between sRAGE levels and acute renal failure as well as mortality could also be assessed [193]. After colectomy, sRAGE levels were higher in patients who underwent laparoscopic colectomy than after hand-assisted or open colectomy, probably indicating a protective role in recovery after surgery [194]. As part of a diagnostic panel sRAGE seems to be a useful diagnostic tool for mortality prediction in SIRS and sepsis [195] and in definition of primary graft dysfunction after lung transplantation [196].

### 6.2. Clinical Relevance of Circulating HMGB1 and sRAGE in Malignant Disease

HMGB1 serum baseline levels of 227 patients undergoing gastrointestinal endoscopy differed significantly between the five created subgroups of nonmalignant diseases, high risk (e.g., adenomas), early gastric cancer, advanced gastric cancer and metastasized gastric cancer. HMGB1 serum levels further correlated positively with tumor size, T-stage, N-stage and poor outcome [197]. In patients suffering from hepatocellular carcinoma, HMGB1 serum levels were also significantly elevated compared to patients with chronic hepatitis, liver cirrhosis and healthy controls, being positively correlated to AFP serum levels, TNM-Staging, Edmondson grading and Cancer of the Liver Italian Program Score [198].

In non-small cell lung cancer (NSCLC), HMGB1 levels in sera of 145 patients were significantly higher than in patients with chronic obstructive pulmonary disease (*n =* 77) and 49 healthy donors. Furthermore, there was a positive correlation between HMGB1 serum levels and tumor size and TNM stage [199]. A study on early detection of recurrent cervical squamous cell carcinoma recorded circulating HMGB1 levels to be significantly elevated in 112 patients with recurrent disease when compared to 174 patients with non-recurrent disease and 128 healthy controls, correlating with FIGO stage and negatively with disease-free and overall survival [200]. In patients suffering from advanced pancreatic cancer baseline sRAGE levels were significantly lower than in 28 healthy individuals [201].

Not only in solid tumors but also in children suffering from acute lymphocytic leukemia (ALL), HMGB1 serum levels were significantly higher in the ALL initial treatment group than in healthy controls and children with complete remission. Between healthy control and complete remission no statistical difference was observed [202].

For optimal monitoring of therapy response, reliable markers indicating response or failure as fast as possible are needed. Experimental studies on time-dependence of HMGB1 level elevation showed increased levels in a rat model with glioblastoma multiforme five days after treatment with adenoviral vectors [203], and elevated extracellular HMGB1 levels 48 h after *in vitro* treatment of glioma, melanoma and lung carcinoma cells with temozolomide [146]. Another study on melanoma cells treated with lymphokine-activated killing cells led to detectable HMGB1 levels in cell culture supernatant as early as 4 to 24 h [204]. Interestingly, HMGB1 release into extracellular space seems to be dependent on the type of chemotherapy applied, as tumor cells of various entities treated with alkylating agents melphalane or paclitaxel released HMGB1 measurably after 36 to 40 h, whereas treatment with oxaliplatin led to no significant extracellular elevation [205].

The correlation with therapy response and prognosis was addressed in several studies: In 78 patients with advanced pancreatic cancer under systemic cytotoxic chemotherapy, weekly determined HMGB1 serum levels decreased in progressive and non-progressive patient groups, showing no significant changes at most time points during therapy until first staging after medium 56 days. Pre-therapeutic HMGB1 levels were increased and sRAGE levels decreased as compared with healthy control groups; however, this did not correlate with therapy response either. Interestingly, sRAGE baseline levels were stable in progressive patients while increasing in non-progressive patients. At time of staging at 8 weeks after start of therapy, sRAGE levels were significantly lower in progressive patients than in non-progressive and low pretherapeutic sRAGE values correlated with poor progression-free survival. As the established biomarkers CA 19-9 and CEA had strong predictive and prognostic value, too, HMGB1 and sRAGE alone could not replace them. However, future studies may reveal their use at certain time points, in defined therapies or in marker panels [201].

After local anti-tumor treatment like selective internal radiation therapy (SIRT) or transarterial chemoembolization (TACE), HMGB1 and RAGE also had predictive power and were appropriate markers for therapy response. As local therapies are extremely effective, marker levels were assessed in shorter intervals, namely already 24 and 48 h after treatment. Radiological evaluation of response was determined after one (TACE) to three months (SIRT) [206,207]. Patients suffering from progressive colorectal cancer had significantly higher HMGB1 serum levels 24 h after SIRT compared to non-progressive patients. HMGB1 levels assessed before and 24 h after SIRT further correlated negatively with overall survival. A multivariate analysis on the combination of HMGB1 and CRP even revealed similar prognostic strength as established liver markers in earlier studies [207]. After TACE therapy of primary liver cancer, HMGB1 levels were similar in both response groups, yet sRAGE levels were significantly decreased in progressive patients before and 24 h after TACE [206].

In a study on patients with localized breast cancer undergoing neoadjuvant chemotherapy, pre-therapeutic sRAGE levels were lower in patients than in healthy controls, while HMGB1 levels were similar in both groups. Concerning therapy response, however, baseline HMGB1 levels in patients with no response to therapy were higher and sRAGE levels were lower than in patients with histological partial or complete remission at tumor surgery. Even if these results were only borderline significant, they back the earlier results of the above mentioned studies and may be useful in clinical decision making, as established breast biomarkers CEA and CA 15-3 could not provide any predictive information in this setting [208,209].

In summary, the findings of these clinical studies underline the relevance of circulating HMGB1 and sRAGE for staging, therapy monitoring and prediction in diverse cancer settings. Thereby, high HMGB1 levels before or shortly after initiation of therapy are correlated with poor outcomes, and so are low sRAGE levels before, shortly after, or in the course of antitumor therapy. As this tendency was seen also in non-malignant disease, a sharp differential diagnosis or even their use as screening markers are not probable. However, these two closely linked markers have important abilities and may soon be used in marker panels not only in staging, but also in therapy monitoring, prediction of therapy response and estimation of prognosis.

## 7. Conclusions and Perspectives

HMGB1 and sRAGE exert a multitude of functions in immune modulation in many acute and chronic diseases such as trauma, acute inflammation and autoimmune diseases. In malignant diseases, these two also play an important role in creating a pro-inflammatory microenvironment promoting tumor growth, modulation of effective anti-tumor immune response, angiogenesis and metastasis. Being present in measurable amounts in blood circulation, HMGB1 and sRAGE can easily be used as clinical biomarkers in prediction, prognosis, therapy monitoring and staging of cancer diseases. However, circulating levels show a wide range, even in healthy persons, and HMGB1 and sRAGE are not specific for certain diseases. Therefore, they are not appropriate for screening or differential diagnosis. Yet pre-therapeutic high levels of HMGB1 and low levels of sRAGE were shown to bear non-favourable predictive and prognostive information, as well as levels determined early after initiation of therapy or in the course of therapy. Due to their involvement in the regulation of the immune system, a special focus of future research will be set on the relevance of circulating HMGB1, and sRAGE, and their subclasses for stratification and monitoring of new immune modulating therapies in cancer and autoimmune diseases.
